# Supplementary material for: Eye-head coordination and dynamic visual scanning as indicators of visuo-cognitive demands in driving simulator
Source: PLoS One. 2020 Dec 31;15(12):e0240201. doi: 10.1371/journal.pone.0240201 (PMC7774948; doi:10.1371/journal.pone.0240201)
Supplement: Attachment — Submitted filename: Response_reviewers_final.docx [file pone.0240201.s001.docx]

Reviewer #1: Generally, the manuscript is well written. However, revisions are required before acceptance.

**We would like to thank reviewer #1 for the insightful comments which allowed us to clarify some points.**

1. In the experiment, participants were asked to wear contact lenses with varying degrees of defocusing to achieve visual degradation. The manuscript did not specify the extent to which they affected the increase in drivers' visual demands. No significant difference between the low-degradation and high-degradation groups was observed in the study, assuming that the participants could compensate to some extent. This is not rigorous; is there more detail.

**As mentioned in the experimental procedure (L221-224), we measured the visual degradation induced by the contact lenses as a reduction in visual acuity following myopic defocus (4/10 at 3m and 4/10 at 1m20 for the lower and higher degradation groups, respectively). Furthermore, several arguments can be put forward to show that our visual manipulation was present:**

- **The task was designed to be visually demanding since participants had to switch between far and intermediate viewing distances, therefore involving changes in tonic vergence and the cross-coupling between accommodation and vergence (although not directly measured in this specific study but see discussion, L504-512).**
- **Moreover, there is some evidence suggesting that blurred vision can have an impact on cognitive functions (L512-523).**
- **Another paper of ours investigated the driving behavior in the same participants and revealed differences between the two levels of visual degradation. We showed that some driving parameters were more affected in the higher compared to the lower degradation group (manuscript currently in revision for PLoS ONE as well).**
- **After careful data inspection, we had to do some minor adjustments in the computation of entropy measures and update the output of statistical tests (see results, L364-385 and discussion, L458-461). In contrast to what has been reported in the original version, we found a difference in eye entropy between the higher and lower degradation groups (p = 0.040 in the current version; p = 0.075 in the previous version). We also discussed potential factors that might explain why we had very few differences between both experimental groups (L526-539).**

2. 21 participants were selected for the experiment. The degree of astigmatism and other factors also have an impact on the experimental results, such as affecting the anchoring of eye tracker, which was not considered in the experiment.

**When recruiting, we excluded participants with high degree of myopia, hyperopia, astigmatism and anisometropia. Details have been provided in the methods section (L154-155). A specific calibration procedure was also performed to ensure the precision of eye- and gaze-tracking (see also point #4). Hence, we are confident that the degree of astigmatism had no or negligeable impact on our experimental results.**

3. The minimum gaze duration of the eye is 120 ms, therefore, eye and gaze data were divided into time bins of 120 ms. However, whether it is reasonable to divide the head movement data into a time bins of 800 ms? There's no basis here. And the coupling between data is questionable. In the discussion section of experimental results, it is found that drivers focus on the road ahead, assuming that they complete the auxiliary tasks faster than expected, which is lack of experimental verification. Therefore, there should be rigorous consideration in this aspect, which is also helpful for analysis.

**We thank the reviewer for pointing out this mistake from our side. In fact, the head data was divided into time bins of 120 ms as well in order to get comparable entropies between the eye, the gaze and the head, as we mentioned in the discussion of the original manuscript (L454-456). We have rectified the methods section accordingly (L258-259).**

**As for the coupling between data (which we believe refers to the eye-head coordination in Figs 4 and 5), it was done based on the sampling rate which was the same for eye- and head-tracking (i.e., 120 Hz). This computation is therefore independent of the time bins used to compute entropies. In addition, we used rigorous methods of synchronization and calibration in 3D space (L191-201) and we are therefore confident with respect to the robustness of our results.**

**This study was designed to incorporate a more naturalistic driving task. As such, participants were instructed to drive as they would normally do in real life and maintain a constant speed of 90 km/h. Moreover, no cue was given as to when the auxiliary GPS task started so that participants were free to look at the GPS device whenever they felt it was safe or adequate to do so, while still driving. That is why we provided participants with a relatively large time window (6 seconds) to answer. One could assume that, because gaze distributions are centered on the road ahead, participants might have ignored the auxiliary task and focused on the driving task instead. However, the success rates observed for the auxiliary task are high (over 70%) in both optimal and degraded vision (information added to the manuscript, L286-290), thus refuting this hypothesis. Alternatively, it could be that participants completed the task in less than 7 seconds and focused on the road again or they did multiple switches between the primary driving task and the auxiliary GPS task.**

4. According to the density distribution of the yaw/pitch Angle data of the driver's head, eyes and gaze, it is not precise enough to infer the location of the driver's gaze, which is two-dimensional. Different drivers test at different locations of the equipment, that is, there is a big difference in the original position in the spatial coordinate system, there is an individual difference in the projected position in front of them.

**We agree with the reviewer that the tracking of eye and head movements, as well as the estimation of gaze rotations require a specific calibration and spatial coordinate transformations. Moreover, gaze position was inferred in 3 dimensions based on the eye, head and simulator positions in the world reference frame. We have provided a description of the calibration in the methods (L191-201).**

Reviewer #2: The topic is worthy of investigating and the conclusion is useful. The paper is overall well written and structured. There is one minor requested revision to the current paper, there literature review should be exhaustive and include the following driving simulator related studies:

**We would like to thank reviewer #2 for their feedback and suggestions.**

[1] Examining the safety of trucks under crosswind at bridge-tunnel section: A driving simulator study, Tunnelling and Underground Space Technology, 2019, 92, 103034. https://doi.org/10.1016/j.tust.2019.103034

[2] Examining the influence of decorated sidewaall in road tunnels using fMRI technology, Tunnelling and Underground Space Technology, Volume 99, 2020, <https://doi.org/10.1016/j.tust.2020.103362>

**The first paper addresses the question of truck safety in bridge-tunnel sections by describing the directional stability and the lateral displacement of the moving vehicle in response to external environmental perturbations (crosswind), in a driving simulator. The results shed light on the hazards of driving in these specific road sections. However, we are confused as to how it relates to our present findings; unless the reviewer wants to clarify why and where in the manuscript it should be appropriate to cite this reference.**

**The second paper investigates the influence of decorated tunnel sidewalls on brain activity through fMRI. Although we acknowledge the work done in this study and its importance regarding safety in monotonous tunnel driving environment, the driving scenarios used in our study were open roads and designed to be realistic (i.e. non monotonous environment). We are therefore skeptical about the relevance of including this reference to our manuscript, but we would be happy to get further details from the reviewer.**
